# N-terminal pro-brain natriuretic peptide as an early prognostic factor in cancer patients developing septic shock

**DOI:** 10.1186/cc5721

**Published:** 2007-03-14

**Authors:** Djamel Mokart, Antoine Sannini, Jean-Paul Brun, Marion Faucher, Didier Blaise, Jean-Louis Blache, Catherine Faucher

**Affiliations:** 1 Department of Anesthesiology and Intensive Care Unit, Paoli-Calmette Institute, 232 bd Sainte Marguerite, 13273 Marseille Cedex 9, France; 2 Department of Hematology, Paoli-Calmette Institute, 232 bd Sainte Marguerite, 13273 Marseille Cedex 9, France

## Abstract

**Introduction:**

The overall prognosis of critically ill patients with cancer has improved during the past decade. The aim of this study was to identify early prognostic factors of intensive care unit (ICU) mortality in patients with cancer.

**Methods:**

We designed a prospective, consecutive, observational study over a one-year period. Fifty-one cancer patients with septic shock were enrolled.

**Results:**

The ICU mortality rate was 51% (26 deaths). Among the 45 patients who benefited from transthoracic echocardiography evaluation, 17 showed right ventricular dysfunction, 18 showed left ventricular diastolic dysfunction, 18 showed left ventricular systolic dysfunction, and 11 did not show any cardiac dysfunction. During the first three days of ICU course, N-terminal pro-brain natriuretic peptide (NT-proBNP) levels were significantly higher in patients presenting cardiac dysfunctions compared to patients without any cardiac dysfunction. Multivariate analysis discriminated early prognostic factors (within the first 24 hours after the septic shock diagnosis). ICU mortality was independently associated with NT-proBNP levels at day 2 (odds ratio, 1.2; 95% confidence interval, 1.004 to 1.32; *p *= 0.022). An NT-proBNP level of more than 6,624 pg/ml predicted ICU mortality with a sensitivity of 86%, a specificity of 77%, a positive predictive value of 79%, a negative predictive value of 85%, and an accuracy of 81%.

**Conclusion:**

We observed that critically ill cancer patients with septic shock have an approximately 50% chance of survival to ICU discharge. NT-proBNP was independently associated with ICU mortality within the first 24 hours. NT-proBNP could be a useful tool for detecting high-risk cancer patients within the first 24 hours after septic shock diagnosis.

## Introduction

The overall prognosis of critically ill patients with cancer has improved during the past decade [1,2]. Reports concerning critically ill patients with malignancies admitted to the intensive care unit (ICU) include a wide range of causes, including septic shock. In this population, mortality rates tend to be approximately 50% when septic shock is present [2,3]. Despite the presence of life-threatening factors such as neutropenia or bone marrow transplantation, prognostic factors in the development of septic shock in patients with cancer are related mainly to the importance of organ dysfunctions [2]. Septic shock is commonly associated with myocardial dysfunction [4], for which accurate evaluation at bedside is not easy. In fact, patients with septic shock show reversible left ventricular systolic dysfunction (LVSD) often masked by a concomitant elevation in the cardiac index [5]. Cardiac troponins and natriuretic peptides are commonly used for diagnosis and risk stratification in patients with acute coronary syndrome and congestive heart failure. Their prognostic and diagnostic relevance is still under investigation in patients with septic shock [6]. The pro-brain natriuretic peptide (proBNP) is produced by atrial and ventricular myocytes in response to wall stress [7]. On secretion, the precursor molecule proBNP is split into N-terminal proBNP (NT-proBNP) and the physiologically active C-terminal peptide comprising 32 amino acids (BNP). Plasma BNP and NT-proBNP measurements are useful in diagnosing systolic [8] and diastolic [9] heart failure, and their concentrations have been shown to be predictive of mortality in patients with septic shock [10]. Moreover, in patients with cancer, a persistent increase of NT-proBNP early after administration of high-dose chemotherapy is strongly associated with the development of cardiac dysfunction [11]. In patients with cancer, septic shock remains associated with a high risk of death, and early evaluation and treatment are essential for maximizing the chance of recovery. The aim of this study was to investigate early prognostic factors in patients with cancer who developed septic shock.

## Materials and methods

The study was conducted prospectively over a 13-month period in a cancer hospital. ICU admission occurred between 1 December 2004 and 16 December 2005. After receiving approval from our institutional ethics committee and obtaining the informed consent of the patients or next of kin, we performed the study in 51 consecutive adult cancer patients with medical septic shock. Septic shock was defined according to the criteria of the American College of Chest Physicians/Society of Critical Care Medicine Consensus Conference [12,13]: (a) clinical evidence of infection, (b) tachycardia (more than 90 beats per minute), (c) tachypnea (more than 20 breaths per minute) or the need for mechanical ventilation, (d) refractory hypotension defined by a sustained decrease in systolic blood pressure below 90 mm Hg despite fluid replacement (40 ml/kg) or the use of a vasopressor to maintain systolic blood pressure above 90 mm Hg, and (e) evidence of inadequate organ function or perfusion within 12 hours of enrollment, as manifested by at least one of the following syndromes: acute alteration of mental status, arterial hypoxemia (PaO_2_/FiO_2 _[arterial partial pressure of oxygen/fraction of inspired oxygen] of less than 280), plasma lactate concentrations above the normal range or metabolic acidosis, oliguria defined by a urine output of less than 0.5 ml/kg per hour, or disseminated intravascular coagulation.

Exclusion criteria included age of less than 18 years and chronic illnesses known to increase NT-proBNP levels, such as chronic heart insufficiency defined by a left ventricular ejection fraction (LVEF) of less than 45% or valvular heart disease, chronic obstructive lung disease, pre-existent renal insufficiency (history of serum creatinine of more than 180 μmol/l before the onset of septic shock), and brain disorders.

Patients with septic shock were systematically admitted to the ICU once the diagnosis was performed. They came from hematology or oncology units. All patients benefited from broad-spectrum antibiotic treatment (betalactamin plus an aminoside or a quinolone plus a glycopeptide) immediately after the initial clinical evaluation. All patients also benefited from standard supportive care for the shock according to the Surviving Sepsis Campaign [14] once they had been managed by the physician. Fluid expansion (using crystalloids or colloids) was firstly used to increase blood pressure. Then, the use of epinephrine, norepinephrine, and/or dobutamine was decided by the physician in charge of the patient. Stress-dose steroids were routinely administered in our patients presenting septic shock. Hemodynamic exploration, using echocardiography or a Swan-Ganz catheter, was routinely performed in all patients presenting septic shock.

Standard biological data were prospectively collected during the ICU stay. The following clinical data were prospectively collected during the ICU stay: age and gender; chronic health status as evaluated using the Charlson comorbidity index [15]; characteristics of the malignancy, including the number of previous courses of chemotherapy and current status (complete or partial remission); neutropenia (white blood cell count of less than 1,000 leukocytes per cubic millimeter and/or neutrophil count of less than 500 per cubic millimeter); infection category (clinically documented infection or microbiologically documented infection); severity-of-illness scores using Simplified Acute Physiology Score II at admission [16]; and Logistic Organ Dysfunction (LOD) score on day 1 (day of septic shock diagnosis) and day 2 [17]; the presence of persistent organ dysfunctions of more than 48 hours according to the LOD score; therapeutic interventions, including vasopressor use, inotrope use, mechanical ventilation, duration of mechanical ventilation, renal replacement therapy, duration of renal replacement, length of ICU stay; and time from sepsis diagnosis to ICU admission and time from ICU admission to septic shock diagnosis and ICU mortality. Early prognostic factors were defined as easy-to-collect, clinical and biological factors available at bedside within the first 24 hours after the septic shock diagnosis (days 1 and 2).

### Echocardiographic evaluation

In our ICU, right catheterization is rarely performed and transthoracic echocardiography (TTE) is commonly used to assess the hemodynamic status of critically ill patients. TTE was performed daily during the septic shock course but not necessarily at the same time as the NT-proBNP measurement. Forty-five (88%) patients received daily TTE evaluation of left and right ventricular function, and six patients could not be evaluated because of their bad echogenicity. TTE was performed using a commercially available ultrasound machine, Acuson 'Cypress' (Acuson Corp., part of Siemens AG, Munich, Germany). Conventional M-mode and two-dimensional echocardiographic measurements were performed according to the guidelines of the American Society of Echocardiography [18,19]. In accordance with Simpson's method of estimation of ejection fraction, the LVEF was divided into two groups: normal or slightly reduced (ejection fraction of greater than 45%) and reduced (ejection fraction of less than 45% = LVSD). Left ventricular diastolic dysfunction (LVDD) (that is, impaired relaxation, pseudonormal pattern, and restrictive pattern) was suggested by alterations in the Doppler mitral flow (ratio of early filling wave velocity [E] to atrial filling wave velocity [A]) and/or alterations in myocardial early diastolic velocity (Em) and myocardial atrial velocity (Am) by tissue Doppler at lateral mitral annulus resulting in modifications of the Em/Am ratio [20,21] (Table 1). The mitral inflow velocity was recorded from the apical four-chamber view with the pulsed-wave Doppler sample volume positioned between the tips of the mitral leaflets during diastole. E and A of mitral inflow were obtained. Septic right ventricular dysfunction (RVD) was defined as suggested by Vieillard-Baron and colleagues [4]. Briefly, RVD was defined as a septal dyskinesia and a dilatation of the right ventricle (end-diastolic diameter of more than 30 mm from the parasternal view or the right ventricle appearing larger than the left ventricle from the subcostal or apical view) or the association of right ventricle dilatation and pulmonary arterial hypertension (PAH). PAH was defined as a pulmonary artery systolic pressure (PASP) of more than 45 mm Hg. The echocardiographic assessment of the PASP was made by the modified Bernoulli equation (P = 4V^2 ^+ right atrial pressure, where P was the peak pressure gradient between the right atrium and the right ventricle, V was the peak velocity of the tricuspid regurgitant jet, and right atrial pressure was assimilated to central venous pressure). Cardiac dysfunctions were defined as the presence of LVSD, LVDD, or RVD for more than 48 hours.

**Table 1:** Doppler echocardiographic pattern in relation to the diagnosis and the grading of left ventricular diastolic dysfunction

		Left ventricular diastolic dysfunction		
		
Parameter	Normal pattern	Pattern of abnormal relaxation	Pseudonormal pattern	Restrictive pattern
E/A	> 1	< 1	1–2	> 2
Ea (cm/s)	> 8	< 8	< 8	< 5
E/Ea	< 8			> 16

E/A, ratio of transmitral early filling wave velocity to atrial filling wave velocity; Ea, myocardial early diastolic velocity by tissue Doppler at lateral mitral annulus; E/Ea, ratio of transmitral early diastolic velocity to myocardial early diastolic velocity of lateral mitral annulus by tissue Doppler.

### Assay for N-terminal pro-brain natriuretic peptide

Plasma levels of NT-proBNP were measured at septic shock diagnosis (day 1) and on days 2 and 3. The venous blood samples were collected in 10-ml vacutainers containing lithium-heparin (Becton Dickinson Biosciences, San Jose, CA, USA), which were placed on ice and transported to our laboratory for immediate assay. The samples were centrifuged at 3,000*g *for 10 minutes. NT-proBNP values were determined by an electrochemiluminescence sandwich immunoassay with an Elecsys 2010 instrument (Roche Diagnostics GmbH, Mannheim, Germany). The interassay coefficients of variation are 3.2% at 175 pg/ml, 2.9% at 355 pg/ml, and 2.6% at 1,068 pg/ml [22]. The analytical range extends from 20 to 35,000 pg/ml. Upper values are obtained by diluting samples. The total duration of the assay was 18 minutes.

### Assay for cardiac troponin I

Plasma levels of cardiac troponin I (cTnI) were measured on days 1, 2, and 3. The venous blood samples were collected in 10-ml vacutainers containing lithium-heparin (Becton Dickinson), which were placed on ice and transported to our laboratory for immediate assay. The samples were centrifuged at 3,000*g *for 10 minutes. For the measurement of cTnI, we used a sandwich immunoassay test (Dade Behring, Inc., Deerfield, IL, USA) on the Dimension RxL analyzer(Dade Behring, Newark, DE, USA). The upper limit of normal for cTnI was set at 0.2 μg/l. The values of interassay imprecision were 7.6% at 0.27 μg/l and 8.1% at 28.3 μg/l [23].

### Statistical analysis

Categorical data are presented as number (percentage). Quantitative data are presented as median (25th to 75th percentiles). Statistical analysis was performed using SPSS software (version 12.0; SPSS Inc., Chicago, IL, USA). Univariate analysis was conducted to determine prognostic factors for the occurrence of ICU death after septic shock. All the parameters collected in Table 2 were considered for univariate analysis. Chi-square tests or Fisher exact tests were used for qualitative variables. The Mann-Whitney test was used for continuous variables. To investigate the correlations between two single variables, Spearman rank correlation was performed. A multivariate analysis was conducted to quantify the respective role of each variable on the occurrence of ICU death. A stepwise logistic regression was performed (backward method). The explanatory variables included in the logistic regression were variables identified as potential prognostic factors by the univariate analysis (cutoff *p *< 0.05). To avoid increasing the risk of collinearity for the same variables measured at days 1 and 2 (LOD and NT-proBNP), we decided to include only the most significant of them in the logistic regression analysis because these variables are often highly correlated [24,25]. The condensed model was presented with odds ratio and 95% confidence interval (CI). In regard to NT-proBNP, discrimination was assessed using the area under the receiver operating characteristic (ROC) curve to evaluate how well the model distinguished patients who died in the ICU. Cumulative survival rates in patients with septic shock according to the cutoff value of NT-proBNP are presented by Kaplan-Meier diagram, and differences among groups were tested by the log-rank test. The required significance level was set at a *p *value of less than 0.05.

**Table 2:** Early prognostic factors for ICU mortality, univariate and multivariate analysis

	Survivors (*n *= 25)	Non-survivors (*n *= 26)	*p *value
NT-proBNP in pg/ml on day 1	3,414 (754–9,005)	7,939 (4,495–33,662)	0.0015
NT-proBNP in pg/ml on day 2	3,145 (990–6,490)	13,091 (8,132–40,627)	< 0.0001
cTnI in μg/l on day 1	0.05 (0.0–0.168)	0.155 (0.090–0.290)	0.03
cTnI in μg/l on day 2	0.04 (0.020–0.210)	0.125 (0.105–0.190)	0.08
LOD score on day 1	7 (6–9)	10 (9–12)	0.0002
LOD score on day 2	5 (4–6)	8 (5–9)	0.0009
SAPS II at ICU admission on day 1	50 (41–60)	48 (35–82)	0.49
Lactate in mmol/l on day 1	1.8 (1.3–2.9)	3.0 (1.6–6.8)	0.14
Lactate in mmol/l on day 2	1.8 (1.3–2.6)	2.6 (1.4–4.8)	0.22
Charlson comorbidity index	2 (2–4)	3 (2–4)	0.76
Age in years	58 (47–68)	55 (51–70)	0.65
Male gender	16 (64%)	16 (61.5%)	0.9
Time from sepsis to ICU admission in days	2 (0–5)	3 (0–9.75)	0.38
Neutropenia	8 (32%)	12 (46%)	0.4
Anthracyclin treatment	14 (56%)	14 (54%)	0.8
Autologous HSCT	0 (0%)	6 (23%)	0.023
Allogeneic HSCT	2 (8%)	3 (11.5%)	0.9
Lymphoma	6 (24%)	9 (35%)	0.54
Acute leukemia	10 (40%)	8 (31%)	0.56

Multivariate logistic regression	*P *value	Odds ratio	95% CI
NT-proBNP in pg/ml on day 2	0.022	1.2	1.004–1.32

Data are expressed as number (percentage) or as median (25th–75th percentiles). *p *< 0.05 was considered significant. CI, confidence interval; cTnI, cardiac troponin I; HSCT, hematopoietic stem cell transplantation; ICU, intensive care unit; LOD, Logic Organ Dysfunction; NT-proBNP, N-terminal pro-brain natriuretic peptide; SAPS II, Simplified Acute Physiology Score II.

## Results

Our study included 51 patients, 32 men (63%) and 19 women (37%). Patients were a median of 56 (50 to 68) years old. The characteristics of the patients are reported in Table 3. Eighteen patients (35%) presented acute leukemia, 16 patients (31%) presented a lymphoma, 11 patients (21%) presented a solid tumor, and 6 patients (12%) presented myelodysplastic syndrome. Twenty patients (39%) were neutropenic, and 11 received hematopoietic stem cell transplantation (HSCT) (21%). Forty-three patients (84%) benefited from recent chemotherapy; of these, 28 (65%) were treated with anthracyclins. During the ICU course, respiratory dysfunction was present in 48 patients (94%), neurologic dysfunction was present in 16 patients (31%), hepatic dysfunction was present in 27 patients (53%), and renal dysfunction was present in 26 patients (51%). The ICU mortality rate was 51% (26 deaths).

**Table 3:** Characteristics of the patients

	Number (percentage) or median (25th–75th percentiles)
Age in years	56 (50–68)
Male gender	32 (62%)
SAPS II at ICU admission	50 (35–71)
LOD score on day 1	10 (6–10)
Time from sepsis to ICU admission in days	2 (0–6)
Time from ICU admission to septic shock in days	0 (0–1)
Norepinephrine treatment	46 (89%)
Epinephrine treatment	23 (45%)
Dobutamine treatment	15 (29%)
Mechanical ventilation	49 (96%)
Duration of mechanical ventilation in days	11 (7–20)
Renal replacement	24 (47%)
Duration of renal replacement in days	1 (0–7)
Type of infection	
Pulmonary sepsis	28 (55%)
Abdominal sepsis	8 (15.7%)
Urinary sepsis	1 (2%)
Isolated bacteremia	14 (2 7.5%)
Microorganism	
Gram-negative bacilli	18 (35.3%)
Gram-positive cocci	13 (25.5%)
Fungus	9 (17.5%)
Viruses	1 (2%)
Not documented	10 (19.6%)
ICU length of stay in days	18 (10–28)
ICU mortality	26 (50.1%)

ICU, intensive care unit; LOD, Logic Organ Dysfunction; SAPS II, Simplified Acute Physiology Score II.

The univariate analysis showed that NT-proBNP levels, LOD score, and cTnI levels were significantly associated with ICU mortality. Among the characteristics of the malignancies, autologous HSCT was the only parameter associated with ICU mortality (Table 2). The multivariate analysis showed that only NT-proBNP level at day 2 was an early independent factor of ICU mortality (Table 2). When gender and age, which are known to influence NT-proBNP, were introduced in the stepwise logistic regression, the results were the same. Other factors such as cTnI or lactates were not independently associated with ICU mortality.

**Figure 1:**
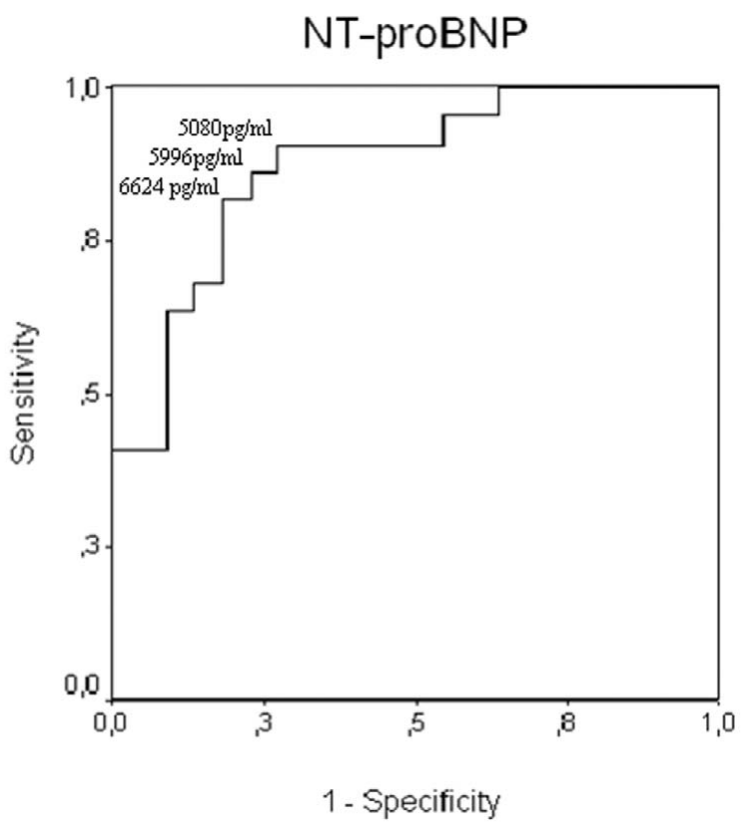
Receiver operating characteristic curve for N-terminal pro-brain natriuretic peptide (NT-proBNP). Receiver operating characteristic curve for determination of NT-proBNP levels as a graphic representation of the relationship between sensitivity (true-positive rate) and 1 - specificity (false-positive rate). The area under the curve for NT-proBNP (area under the curve, 0.87; *p *< 0.0001) summarizes the capacity of NT-proBNP as a valuable predictor of mortality of patients with septic shock.

**Figure 2:**
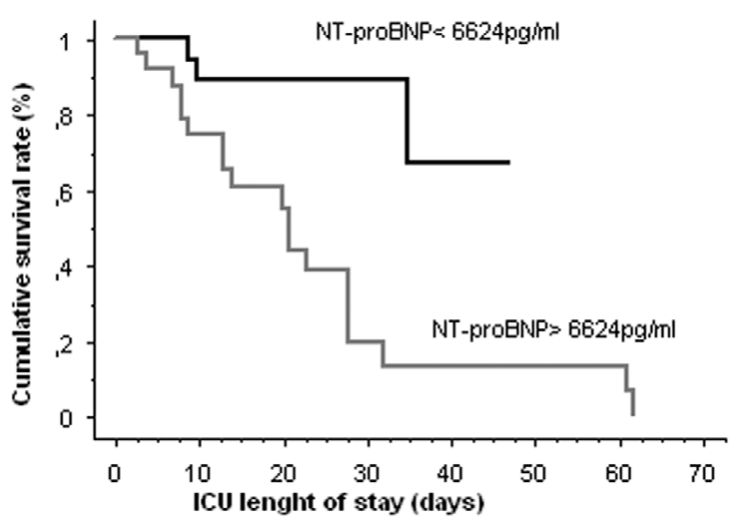
Kaplan-Meier curve for N-terminal pro-brain natriuretic peptide (NT-proBNP) at the cutoff value of 6,624 pg/ml. Kaplan-Meier analysis estimates the rate of death within intensive care unit (ICU) stay among septic shock patients according to NT-proBNP values above or below 6,624 pg/ml (cutoff value as determined by receiver operating characteristic curve analysis). The differences between the two groups were significant (*p *= 0.0011 by the log-rank test).

On day 1, NT-proBNP levels were positively correlated to LOD score (*r *= 0.39, *p *= 0.008) and cTnI levels (*r *= 0.567, *p *= 0.0005). On day 2, NT-proBNP levels were positively correlated to LOD score (*r *= 0.565, *p *= 0.0002) and cTnI levels (*r *= 0.480, *p *= 0.004). An ROC curve was constructed taking into account NT-proBNP levels on day 2 (Figure 1). An NT-proBNP level of greater than 6,624 pg/ml predicted ICU mortality with a sensitivity of 86%, a specificity of 77%, a positive predictive value of 79%, a negative predictive value of 85%, and an accuracy of 81%. Area under the curve (AUC) was 87% (*p *< 0.0001, 95% CI 0.768 to 0.976). Kaplan-Meier analysis (Figure 2) estimates the rate of death within ICU stay among patients with septic shock according to NT-proBNP values above or below 6,624 pg/ml (cutoff value as determined by ROC curve analysis). Differences between the two groups were significant (*p = *0.0011 by the log-rank test).

In the non-survivors and during the first three days of ICU course, NT-proBNP levels were highest in patients previously treated with anthracyclines compared to patients without anthracycline treatment: 33,662 pg/ml (9,410 to 85,015) versus 5,275 pg/ml (2,953 to 7,939) (*p *= 0.003), respectively, on day 1 and 37,435 pg/ml (11,348 to 93,867) versus 10,070 pg/ml (6,157 to 13,520) (*p *= 0.012), respectively, on day 2. In the survivors and during the first three days of ICU course, NT-proBNP levels were similar in patients previously treated with anthracyclines and in patients without anthracycline treatment: 3,498 pg/ml (768 to 8,425) versus 1,396 pg/ml (886 to 8,866), respectively, on day 1 and 4,041 pg/ml (1,246 to 8,195) versus 2,537 pg/ml (914 to 4,170), respectively, on day 2.

Among the 45 patients who benefited from TTE evaluation, 17 showed RVD, 18 showed diastolic dysfunction, 18 showed systolic dysfunction, and 11 did not show any cardiac dysfunction (no cardiac dysfunction group). On day 2, NT-proBNP level was higher in patients presenting cardiac dysfunction but was not higher on day 1 (Table 4). During the ICU course, only the occurrence of RVD was significantly associated with ICU mortality. The same, but non-significant, trend was observed when LVDD was present (Table 5).

**Table 4:** Comparisons of NT-proBNP levels according to the occurrence of cardiac dysfunctions during the intensive care unit course

	RVD (*n *= 17)	LVSD (*n *= 18)	LVDD (*n *= 18)	NCD (*n *= 11)
NT-proBNP in pg/ml on day 1	6,138 (3,530–16,458)	6,214 (3,721–30,409)	9,001 (4,000–33,167)	4,220 (488–17,371)
NT-proBNP in pg/ml on day 2	7,685 (3,965–23,433)^a^	9,083 (3,860–22,784)^a^	11,254 (4,067–37,804)^a^	914 (447–12,545)

^a^*p *< 0.05 compared to the no cardiac dysfunction (NCD) group. Data are expressed as median (25th–75th percentiles). The number of patients in whom NT-proBNP was measured on day 2 was the same as on day 1 (no death on day 1). LVDD, left ventricular diastolic dysfunction; LVSD, left ventricular systolic dysfunction; NT-proBNP, N-terminal pro-brain natriuretic peptide; RVD, right ventricular dysfunction.

**Table 5:** Characteristics of cardiac dysfunctions among patients who benefited from transthoracic echocardiography evaluation (*n *= 45) during the intensive care unit course

	Survivors (*n *= 23)^a^	Non-survivors (*n *= 22)^a^	*p *value
Right ventricular dysfunction	5 (22%)	12 (54.5%)	0.03
Left ventricular systolic dysfunction	9 (39%)	9 (40.1%)	0.9
Left ventricular diastolic dysfunction	6 (26%)	12 (54.5%)	0.07
No cardiac dysfunction	8 (34.5%)	3 (13.6%)	0.16

^a^Data are expressed as number (percentage).

## Discussion

This study was conducted to determine early prognostic factors in the development of septic shock in patients with cancer. We found that the ICU mortality in such patients was similar to that observed in the general population. After full life support management, NT-proBNP on day 2 was an early independent factor of ICU death.

Septic shock is often accompanied by pronounced hemodynamic dysfunction. This dysfunction is associated with left ventricular dysfunction, peripheral vasodilatation resulting in systemic hypotension, hyporesponsiveness to vasopressors, and a reduced vascular resistance. Actually, cardiac dysfunction during septic shock can variably be associated with left and right dysfunction [4,5,26] and systolic and diastolic dysfunction [27,28]. In this situation, high levels of NT-proBNP are associated with poor prognosis and severe cardiac dysfunction [10,29]. Patients treated with doxorubicin chemotherapy exhibit high levels of natriuretic peptides. This may be related to a severe impairment of both systolic and diastolic dysfunction [30]. Among the study group, 45 patients received echocardiographic evaluation and 34 (75.5%) of them showed cardiac dysfunction. Eighteen (40%) of them showed LVSD, 18 (40%) showed LVDD, and 17 (38%) showed RVD. A striking finding from this study is that the cutoff value of NT-proBNP to detect high-risk patients in the first 24 hours was two times lower in patients with cancer than in the general population [10]. However, the NT-proBNP value chosen by Roch and colleagues [10] was the highest value of four measurements performed during the first 48 hours, which could account, in part, for the discrepancies with our results. Nevertheless, our results also suggest that, in the context of septic shock, patients with cancer are more vulnerable to cardiomyocyte stretch than the general population. Fifty-five percent of our patients were treated with a chemotherapy that included anthracycline before the onset of septic shock. Furthermore, in the non-survivor group, NT-proBNP levels were markedly increased in patients previously treated with anthracycline compared to patients without anthracycline treatment whereas no difference was found in the survivor group. Taken together, our findings presume that the occurrence of septic shock-related cardiac dysfunction could be precociously and dramatically worsened by a pre-existing chemotherapy-induced cardiomyopathy in certain patients with cancer. In addition, we showed that NT-proBNP levels were significantly correlated to elevated levels of cTnI. In accordance with previous reports [31], 62% of our patients showed cTnI levels greater than the threshold of myocardial damage (> 0.1 ng/ml) within the first 24 hours (data not shown). Natriuretic peptides and cardiac troponins provide subtle pieces of information about cardiac dysfunction. Natriuretic peptide release reflects wall stress and thus provides information about the functional status of myocardium, whereas troponin release attests to myocyte injury [6]. Increased levels of both cTnI and NT-proBNP have been found in patients with severe sepsis, and a good correlation between these two markers seems to indicate a relationship between the degree of myocyte damage and functional myocardial impairment [29]. NT-proBNP might be a useful tool for detecting sepsis-induced cardiac dysfunctions. At the cutoff value of 6,624 pg/ml, NT-proBNP within the first 24 hours could permit the detection of the high-risk mortality population in cancer patients presenting septic shock. However, the contribution of NT-proBNP in quantifying the risk of death should be evaluated in larger studies.

At the time of septic shock diagnosis (day 1), the levels of NT-proBNP were high. Moreover, these levels were not associated with the occurrence of cardiac dysfunctions (RVD, LVSD, or LDVD). Interestingly, patients without cardiac dysfunction exhibited equally high levels of NT-proBNP, suggesting mechanisms other than atrial or ventricular wall stress in the release of natriuretic peptides [32]. This could also be explained by the fact that most septic patients exhibit low systemic vascular resistance associated with unloading of the left ventricle; in this situation, myocardial depression can be present whereas echocardiographic assessment may show a preserved LVEF [6,33]. In contrast, on day 2, NT-proBNP levels were associated with the occurrence of cardiac dysfunctions. Indeed, LVSD [34], LVDD [28], and RVD [4] have been found in patients with sepsis and thus might contribute to increased NT-proBNP levels [6]. Among cardiac dysfunctions, only RVD was significantly associated with ICU death. RVD has been demonstrated in septic shock by means of echocardiography [4,26]. In agreement with two previous studies using echocardiography [35,36], we found that RVD was observed with echocardiographic evaluation in 38% of the patients. In septic shock, RVD may be related to intrinsic depression in contractility, acute cor pulmonale produced by an acute increase in pulmonary vascular resistance [26], an increase in airway pressure produced by mechanical ventilation when applied on a depressed right ventricle, or chemotherapy [37]. Hemodynamic management of patients presenting RVD remains controversial [38,39], especially with regard to the use of volume loading, vasopressors, and inotropes. Because these factors may influence NT-proBNP release [6], one can suppose that beyond right ventricular wall stress other vital stimuli may account for NT-proBNP release and influence the outcome.

In the univariate analysis and in agreement with previous data [2], ICU mortality was more closely associated with the markers of organ dysfunctions (LOD score) than with cancer status. Indeed, malignancy and factors reflecting the characteristics of malignancy were not independently associated with ICU mortality. Because the outcome of critically ill allogeneic HCST recipients is poor [40], the lack of a significant association between these patients and ICU mortality may be related to the limited sample size of this subgroup of patients. In the present study, neutropenia was not found to be a predictor of mortality in cancer patients with septic shock. Few recent studies have found neutropenic patients to have mortality rates similar to non-neutropenic patients when septic shock was present [2,3]. We also confirm this trend. During the last few years, the mortality rate of septic cancer patients admitted to the ICU has markedly improved. This may be related to the early admission of septic patients in the ICU for the purpose of providing an early goal-directed therapy as recommended by Rivers and colleagues [41] but also to recent advances in the treatment of hematologic malignancies and solid tumors [41-43]. Our study confirms this trend. In our institution, the ICU admission policy is broad and includes most patients with a 'therapeutic project' regardless of their cancer status. All septic patients admitted to the ICU benefited from standardized supportive care according to the Surviving Sepsis Campaign, including stress-dose steroids and early goal-directed therapy [14,41,44]. In addition, patients considered to benefit significantly from immediate ICU admission were managed systematically and daily by both the intensivist and the hematologist/oncologist until ICU admission. Most of our patients exhibited a pronounced thrombopenia, and only one of them benefited from treatment with human recombinant-activated protein C [45]. Despite this inconvenience, the mortality rate in this population of patients was promising. Because NT-proBNP may be a valuable early biological marker that allows risk stratification of septic shock in patients with cancer, early detection of high-risk patients could permit us to evaluate new therapeutic options, including human recombinant-activated protein C.

Our study has several limitations. First, we studied only 51 patients with cancer (of whom 26 died), which is not enough to develop a predictive scoring system. Hence, further large studies are required to validate our findings. Second, NT-proBNP is known to be influenced by hemodynamic parameters such as LVEF or left ventricular stroke work index, which could also be prognostic factors. In our study, RVD and LVDD appear as prognostic factors, but because only 45 of our patients (88%) received an echocardiographic evaluation, they could not be analyzed as predictive factors in the multivariate analysis. Third, in our ICU, right catheterization is rarely performed and TTE is commonly used to assess the hemodynamic status of critically ill patients; this explains the lack of hemodynamic data.

## Conclusion

We observed that critically ill cancer patients with septic shock have an approximately 50% chance of survival to ICU discharge. NT-proBNP could be a valuable and useful tool for detecting high-risk cancer patients within the first 24 hours after septic shock diagnosis.

## Key messages

• The ICU mortality in patients with cancer is even more important than in the general population.

• The prognostic value of natriuretic peptides (NT-proBNP) is confirmed here in cancer patients presenting septic shock.

• During the ICU course, (echographic) septic cardiac dysfunction is associated with high levels of NT-proBNP detected in the early phase of septic shock.

## Abbreviations

A = atrial filling wave velocity; Am = myocardial atrial velocity; CI = confidence interval; cTnI = cardiac troponin I; E = early filling wave velocity; Ea = myocardial early diastolic velocity; HSCT = hematopoietic stem cell transplantation; ICU = intensive care unit; LOD = logistic organ dysfunction; LVDD = left ventricular diastolic dysfunction; LVEF = left ventricular ejection fraction; LVSD = left ventricular systolic dysfunction; NT-proBNP = N-terminal pro-brain natriuretic peptide; PAH = pulmonary arterial hypertension; PASP = pulmonary artery systolic pressure; proBNP = pro-brain natriuretic peptide; ROC = receiver operating characteristic; RVD = right ventricular dysfunction; TTE = transthoracic echocardiography.

## Competing interests

The authors declare that they have no competing interests.

## Authors' contributions

DM collected and analyzed the data and reviewed and coordinated the study. AS and MF collected and analyzed the data. J-PB, J-LB, DB, and CF reviewed and coordinated the study. All authors read and approved the final manuscript.
